# DNA Methylation variability among individuals is related to CpGs cluster density and evolutionary signatures

**DOI:** 10.1186/s12864-018-4618-9

**Published:** 2018-04-02

**Authors:** Domenico Palumbo, Ornella Affinito, Antonella Monticelli, Sergio Cocozza

**Affiliations:** 1 Department of Molecular Medicine and Medical Biotechnology (DMMBM), University of Naples “Federico II”, Naples, Italy; 2 Institute for Experimental Endocrinology and Oncology (IEOS) “Gaetano Salvatore”, CNR, Naples, Italy

**Keywords:** Methylation, Variability, Selective pressure, Blood, GERP-RS

## Abstract

**Background:**

In recent years, epigenetics has gained a central role in the understanding of the process of natural selection. It is now clear how environmental impacts on the methylome could promote methylation variability with direct effects on disease etiology as well as phenotypic and genotypic variations in evolutionary processes. To identify possible factors influencing inter-individual methylation variability, we studied methylation values standard deviation of 166 healthy individuals searching for possible associations with genomic features and evolutionary signatures.

**Results:**

We analyzed methylation variability values in relation to CpG cluster density and we found a strong association between them (*p*-value < 2.2 × 10^− 16^). Furthermore, we found that genes related to CpGs with high methylation variability values were enriched for immunological pathways; instead, those associated with low ones were enriched for pathways related to basic cellular functions. Finally, we found an association between methylation variability values and signals of both ancient (*p*-value < 2.2 × 10^− 16^) and recent selective pressure (p-value < 1 × 10^− 4^).

**Conclusion:**

Our results indicate the presence of an intricate interplay between genetics, epigenetic code and evolutionary constraints in humans.

**Electronic supplementary material:**

The online version of this article (10.1186/s12864-018-4618-9) contains supplementary material, which is available to authorized users.

## Background

Although methylation is the major covalent DNA modification that is present across many different species, the complexity of its function is only partially decoded [1]. Methylation is variable and susceptible to environmental changes due to its main function, which is regulating the gene transcription depending on the cell’s needs. Thus, the hypothesis that this modification could persist across generations is counterintuitive, especially if we consider the demethylation process that occurs during the epigenetic reprogramming in the mammal’s embryonic development [2]. However, despite these considerations, there is emerging evidence showing that methylation could be inherited over generations [3]. Indeed, epigenetic alterations could explain the inheritance of some parentally acquired traits that cannot be explained by Mendelian inheritance, DNA mutations or genetic damages [2]. Although the methylation pattern could be trans-generationally inherited, it is important to underline the variable nature of the methylation, mostly because the plasticity of the epigenome is strongly involved in the adaptation to our environment [2]. Indeed, environmental factors could promote epigenetic variation with direct impact both on disease etiology and on origins of phenotypic/genotypic variations involved in evolutionary processes [4]. In other words, methylation helps gene transcription to face environmental changes. If these changes remain stable enough through different generations, it could be beneficial to fix the gene transcription with a new, stable, methylation pattern [3, 5]. So far, no one has successfully described the hidden mechanism that allows the fixation of the differences in DNA methylation between human populations, but the scientific community is apparently moving toward this direction [1, 6, 7]. Actually, it is rather difficult to analyze such methylation variability, mostly because individuals, within the same population, could differ for many parameters (e.g. age, gender, stress, food intake, or health), and all of them could affect the epigenome. In the last decade, the increasing use of Next Generation Sequencing (NGS) and “BeadChip” techniques has produced a huge amount of data on epigenetic analyses, and methylomes of many individuals are available to the scientific community in public databases along with associated information (i.e. gender, age, health status). In this paper we investigated the variability of methylation values of 485,000 CpGs among 166 healthy individuals, exploiting the public data of the Italian section of the European Prospective Investigation into Cancer and Nutrition (EPIC) cohort [8]. It is our opinion that a better knowledge of the variable nature of the methylation could improve our understanding of many biological processes; to this aim, we investigated possible novel sources of methylation variation (henceforward denoted as MV), such as genomic structural landscape, biological pathways and selective pressure.

## Results

### Inter-individual Methylation variability is related to CpG cluster density

**Fig. 1:**
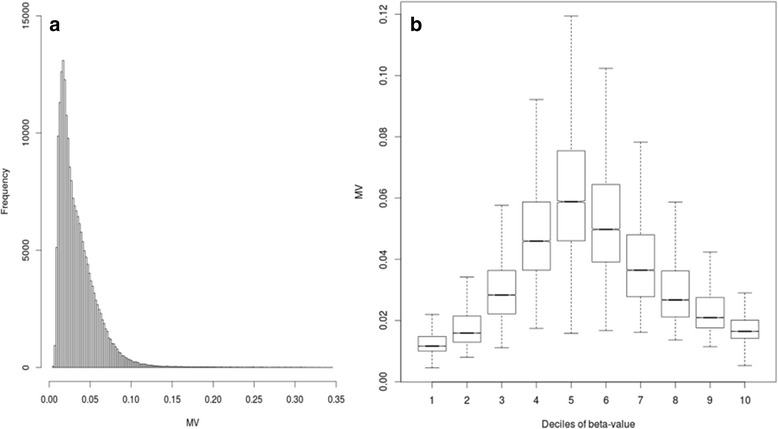
**a** CpGs methylation variation (MV) values distribution in EPIC cohort. **b** Boxplots of CpGs methylation variation (MV) and deciles of methylation values for all CpGs

In this study, we chose the standard deviation as a measure to study CpGs inter-individual methylation variability (MV). Figure 1 shows the distribution of MV values in our population (1A), and the comparison between MV values and methylation beta-values (1B); for the latter, continuous beta-values were binned in deciles.

**Fig. 2:**
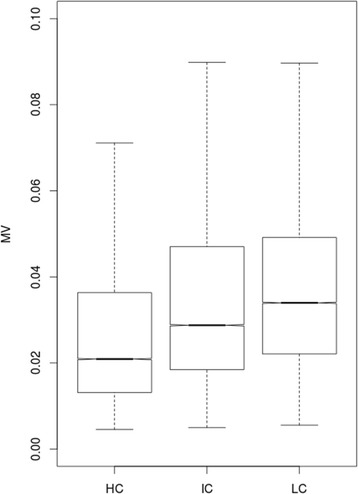
Boxplots of CpGs methylation variation (MV) for CpGs cluster density

We found that MV values were associated with CpGs methylation levels (One-way; *p*-value < 2.2 × 10^− 16^). In particular, higher MV values were observed for CpGs with intermediate methylation levels, while lower MV values were associated with CpGs methylation values near the beta-value limits (0 and 1). To determine if MV values were associated with genomic structural features, we analyzed MV values in relation to CpG cluster density. We classified genomic regions according to their CpGs content into three groups: high-density (HCs, CG content > 55%, Obs/Exp CpG ratio > 0.75 and length > 500 bps), intermediate-density (ICs, CG content > 50%, Obs/Exp CpG ratio > 0.48 and length > 200 bps) and low-density (LCs, non-HC/IC regions) as proposed by Price and colleagues [9]. In our dataset 61,946 CpGs fall in HCs, 72,496 in ICs and 71,999 in LCs. Figure 2 shows the relationship between CpGs MV and their cluster density.

Inter-individual methylation variability showed a strong correlation to CpGs cluster density (One-Way Test, *p*-value < 2.2 × 10^− 16^). Furthermore, CpGs belonging to HCs showed the lowest MV values, while, on the other hand, those belonging to LCs showed the highest ones. Finally, CpGs classified as ICs showed intermediate MV values.

### Functional annotations of genes containing CpGs with different inter-individual Methylation variability

We also investigated whether inter-individual methylation variability was related to specific functional pathways. To attribute to each gene an MV value, we selected 99,376 CpGs located in genomic regions of 3000 base pairs around each Transcription Start Site (for more details see Materials and Methods). Using this approach, we generated a ranked list of genes with MV values of CpGs located in their promoter regions. We used the obtained ranked list to perform a Gene Set Enrichment Analysis (GSEA) [10, 11] using KEGG pathway as gene set. With this approach, we were able to identify 18 KEGG enriched pathways (NOM *p*-value ≤0.01 and FDR q-value ≤0.25) in CpGs with high and 15 with low MV values (Table 1).

**Table 1:** Gene Set Enrichment Analysis output for CpGs with high or low MV

Gene set name	NOM *p*-val	FDR q-val	MV
OLFACTORY TRANSDUCTION	0.000	0.000	High
GRAFT VERSUS HOST DISEASE	0.000	0.000	High
ALLOGRAFT REJECTION	0.000	0.000	High
ASTHMA	0.000	0.000	High
TYPE I DIABETES MELLITUS	0.000	0.000	High
NEUROACTIVE LIGAND RECEPTOR INTERACTION	0.000	0.000	High
AUTOIMMUNE THYROID DISEASE	0.000	0.000	High
INTESTINAL IMMUNE NETWORK FOR IGA PRODUCTION	0.000	0.001	High
METABOLISM OF XENOBIOTICS BY CYTOCHROME P450	0.000	0.018	High
ANTIGEN PROCESSING AND PRESENTATION	0.005	0.032	High
DRUG METABOLISM CYTOCHROME P450	0.004	0.035	High
CELL ADHESION MOLECULES CAMS	0.002	0.033	High
VIRAL MYOCARDITIS	0.009	0.033	High
COMPLEMENT AND COAGULATION CASCADES	0.010	0.032	High
RETINOL METABOLISM	0.008	0.053	High
CYTOKINE CYTOKINE RECEPTOR INTERACTION	0.001	0.052	High
SYSTEMIC LUPUS ERYTHEMATOSUS	0.007	0.054	High
LEISHMANIA INFECTION	0.005	0.064	High
SPLICEOSOME	0.000	0.000	Low
RNA DEGRADATION	0.000	0.000	Low
HOMOLOGOUS RECOMBINATION	0.000	0.000	Low
UBIQUITIN MEDIATED PROTEOLYSIS	0.000	0.000	Low
CELL CYCLE	0.000	0.001	Low
N-GLYCAN BIOSYNTHESIS	0.000	0.004	Low
PARKINSONS DISEASE	0.000	0.006	Low
RNA POLYMERASE	0.003	0.007	Low
LYSINE DEGRADATION	0.000	0.009	Low
HUNTINGTONS DISEASE	0.000	0.013	Low
PROTEASOME	0.004	0.018	Low
TERPENOID BACKBONE BIOSYNTHESIS	0.003	0.017	Low
OXIDATIVE PHOSPHORYLATION	0.000	0.016	Low
AMINOACYL TRNA BIOSYNTHESIS	0.000	0.015	Low
RIBOSOME	0.000	0.018	Low

Interestingly, the enrichment of pathways in CpGs with high MV values was related to immunological processes (e.g. graft vs host disease, allograft rejection, diabetes I disease, autoimmune thyroid response, etc.), while the enrichment of pathways in CpGs with low MV values was related to basic cellular functions (e.g. Spliceosome, RNA degradation, homologous recombination, cell cycle, etc.).

### CpGs under ancient selective pressure show low MV

**Fig. 3:**
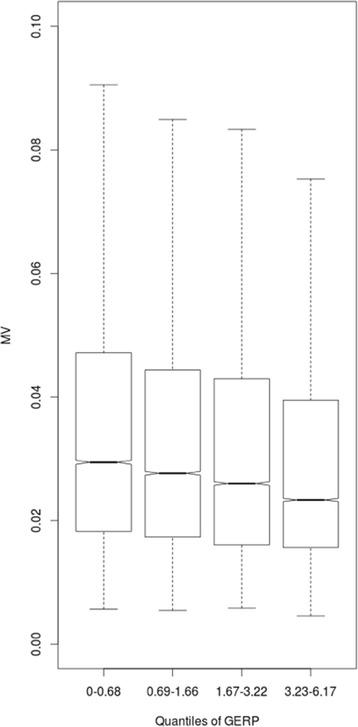
Boxplots of CpGs methylation variation (MV) values (y-axis) and quartiles of Genomic Evolutionary Rate Profiling (GERP) Rejected Score (RS) (x-axis)

We next addressed the role of natural selection in the shaping of inter-individual methylation variability. Ancient and recent selective pressure signals can be detected by analyzing inter- and intra-species diversity. As measure of ancient selective pressure, we used the Genomic Evolutionary Rate Profiling - Rejected substitutions (GERP-RS), a well-accepted method based on sequence conservation among species [12]. GERP-RS scores near zero reveal no substitution deficit and no selective pressure signals, while positive GERP-RS scores represent a substitution deficit, indicating a base conservation during evolution and a possible selective pressure signal. We divided CpGs GERP-RS values into quartiles, and then we calculated MV values for each one (Fig. 3).

Figure 3 shows the presence of an inverse relationship between GERP-RS score and MV (One-Way Test, *p*-value < 2.2 × 10^− 16^). CpGs located in the higher quartile of GERP-RS score (more conserved during evolution) showed the lowest level of MV among individuals.

### CpGs under recent selective pressure show low MV

**Fig. 4:**
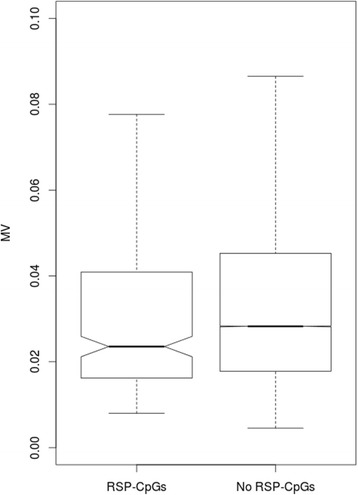
Boxplots of CpGs methylation variation (MV) values between Recent Selective Pressure – CpGs (RSP-CpGs) in TSI and Non Recent Selective Pressure – CpGs (No RSP-CpGs)

Many different measures of recent selective pressure have been proposed [13], but, unfortunately, none of these seems to be fully informative. In order to achieve a more conservative approach, we decided to use a combination of these measures, and additionally filter SNPs that overcame the threshold for each measure (for more details see Materials and Methods) [14]. To this aim, we used data from another Italian population (Tuscans, TSI). By screening the dbPSHP database (http://jjwanglab.org/dbpshp) and applying the above mentioned criteria, we obtained 124 SNPs putatively under recent selective pressure in the Italian population. For each of these SNPs, we identified CpGs localized in a region around 2000 base pair, and we named these CpGs as Recent Selective Pressure CpGs (RSP-CpGs). Figure 4 shows the MV values of RSP-CpGs compared to those who were not classified as recent (No RSP-CpGs).

We found that MV values were lower in regions under recent selective pressure (RSP-CpGs) in comparison with the remaining genomic regions (No RSP-CpGs) (TSI MV mean = 0.027, No RSP-CpGs MV mean = 0.035; bootstrap analysis based on 10,000 Monte Carlo simulations, *p*-value < 1 × 10^− 4^). To confirm this result, we decided to analyze another Caucasian population (CEU). Similarly to what we observed for the Italian population, RSP-CpGs showed lower MV compared to No RSP-CpGs (CEU mean = 0.031, No RSP-CpGs MV mean = 0.035; bootstrap analysis based on 10,000 Monte Carlo simulations, p-value < 3 × 10^− 3^). For additional information about CEU population and bootstrap analyses, see Additional file 1. Finally, we checked whether the 272 TSI RSP-CpGs showed also signals of ancient selective pressure as higher GERP-RS score. We found no differences in terms of GERP-RS scores between RSP-CpGs and No RSP-CpGs (T-test = NS), thus allowing us to speculate that the two selective pressure signatures (ancient and recent), although both showing association with inter-individual methylation variability, are probably reflection of two different phenomena.

## Discussion

Epigenetics is a field of growing interest among scientists involved in different research areas (e.g. cancer, molecular medicine, behavior, development, physiology, morphology, cell biology).

Previous studies investigated the DNA methylation variability in healthy individuals. While the presence of a stochastic component of such variability is indisputable, its relationship with demographic variables such as age [15], gender [16], and smoking behavior [17] is well established. Similarly, the functional impact of this variability on phenotypes has been also demonstrated [18]. DNA methylation variability among healthy individuals has been also hypothesized to influence susceptibility to common diseases, as well as response to drug treatments [6, 19]. In addition, DNA methylation has been also described to differ between ethnic populations [16, 20, 21].

Epigenetics is also achieving a role in our understanding of natural selection and evolution. In a recent review [22], the authors found that about 1% of epigenetics studies were focused on investigating relationships with natural selection and evolution. Most of these papers highlighted the conservation of DNA methylation among species [23, 24], while others emphasized on species-specific DNA methylation [25].

In this study, we analyzed the methylation variability among healthy individuals to investigate its possible relationship with genomic features and evolutionary signatures. To this aim, we exploited the methylation data from the Italian section of the EPIC study [8]. We decided to use, as measure of inter-individual methylation variation (denoted as MV), the standard deviation according to previously published studies [19, 23, 26]. The MV values distribution in our cohort was in agreement with that available in literature [19, 26]. To analyze the possible relationship between methylation variability and genomic features, we decided to use the CpG cluster density, a suitable method to identify biologically-relevant structures [9]. With this approach, we found a strong relationship between inter-individual methylation variability and CpG cluster density, with HC regions showing low levels of CpG methylation variability, while IC and LC regions showed increasingly higher levels. This result is in line with the current knowledge of direct correlation between methylation levels and CpG density [23, 26]. To date, only a few papers have focused on methylation variability. Wagner and colleagues [27] showed a low inter-individual methylation variation in CpGs located near a TSS and highly variable CpGs located far away from it. This finding is in agreement with our results. Indeed, high-density CpG clusters are usually located near a TSS [27]. A possible explanation for this phenomenon could be found in the common hypomethylation across individuals of HC regions, which could cause small inter-individual methylation variations [7]. On the other hand, it should be noted that CpGs with the highest MV, which fall in IC and LC regions, are poorly probed by Illumina 450 K array and tend to fall outside the CpG island [7]. In Acute Lymphoid Leukemia cells it has been found that CpGs falling outside CpG islands show significantly more variation in methylation levels than those falling within the CpG islands [28]. With this knowledge, our results are in agreement with the literature, because the highest number of CpGs falling outside an island belongs to both LC or IC classes [9].

To investigate a possible association between inter-individual methylation variability and functional pathways, we used a GSEA approach. We found an enrichment of pathways related to the immune system in those CpGs with higher inter-individual variability, while we proved an enrichment of pathways related to basic cellular functions in CpGs showing a low inter-individual variability. Previous studies investigated CpGs methylation in three different human populations, showing 439 differential methylated CpGs among groups [6]. Interestingly, genes harboring these CpGs in their promoters are responsible for immune response factors and xenobiotic metabolism. Another study analyzed gene expression from blood samples obtained by 200 healthy controls, and it showed that hypervariable transcripts among individuals were enriched for genes mostly involved in mediating immune-related processes [29]. A possible explanation of these results could be found just in the biology of the blood samples. Since in these samples DNA molecules are extracted from white blood cells, it is reasonable to hypothesize that variable epigenetic signals could partly allow us to face different immunological scenarios, with differences between individuals. Expression of genes involved in the immune response is very variable in a population. Indeed, each individual, comes in contact with different pathogens or xenobiotics during his life, activating in different ways the immune system [30]. In addition, we also examined the inter-individual methylation variability of CpGs that are conserved among species positing that they likely have a biologically relevant function. We found that those CpGs that are more conserved during evolution showed the lowest values of inter-individual methylation variability. Comparing human-mouse methylation, a study showed that methylation correlates, although weakly, with sequence conservation [31]. Analyzing the relationship between DNA methylation, genetics, and expression in fibroblasts, other authors proved that CpGs with low inter-individual methylation variation showed a good degree of sequence conservation [27]. We then tested the hypothesis of the possible relation between recent selective pressure and inter-individual methylation variability among individuals of the same population. We found that inter-individual methylation variability proved to be lower in regions under recent selective pressure compared to the other regions. A possible speculation to explain this finding is that our results could indicate a recent evolutionary fixation of the methylation values. In particular, the inter-individual methylation fixation could indicate both a pure epigenetic fixation or a genetic variation associated with a specific methylation pattern [32]. In the first case, it might be possible that a random methylation pattern could have somehow improved the human adaptation, by providing a favorable phenotype in a specific environment. On the other hand, in the second hypothesis, it could be hypothesized that a favorable genetic mutation could be related to a specific methylation pattern, mostly because it is the only possible for that specific mutation. With this knowledge, it is worth to mention that some authors showed how epigenetic mechanisms can drive genetic changes [33, 34], while other studies proposed that genetic variation could drive DNA methylation differences, and suggested that different methylated CpG sites could work as evolutionarily mediators between the genetic code and the phenotypic variability [6].

## Conclusions

In this study, we investigated novel sources of inter-individual methylation variation such as genomic structural landscape, biological pathways and selective pressure. To this aim, we exploited the public methylation data of the Italian section of the European Prospective Investigation into Cancer and Nutrition (EPIC) cohort [8].

We found that inter-individual methylation variation is strongly correlated to CpG cluster density and signals of both ancient and recent selective pressure. Furthermore, we found that genes related to CpGs with high inter-individual methylation variation were enriched for genes involved in immunological pathways; instead, those associated with low one were enriched for pathways related to basic cellular functions.

Despite the limitation of a small cohort or the use of a single tissue, our results indicate the presence of an intricate interplay between genetics, epigenetic code and evolutionary constraints in humans. Further studies, including longitudinal ones, in different populations are needed to confirm our results. It is our opinion that a better knowledge of the variable nature of the methylation could improve our understanding of many biological processes.

## Methods

### Data preprocessing

Methylome data of 845 participants from the EPIC-Italy cohort were obtained by querying GEO database (GSE51032). The cohort is produced at the Human Genetics Foundation (HuGeF) in Turin, Italy, and contains raw data and normalized methylation values (beta-values) from peripheral blood cells of 188 men and 657 women [8]. All the beta-values from the EPIC project were normalized by the consortium using the same routines [8]. In particular: GenomeStudio software was used for background subtraction and dye bias correction, while batch effects were corrected using COMBAT software [35, 36]. Finally, to test if white blood cell variability could influence the inter-individual methylation variability in our data, we performed a test using the correction described by van Veldhoven et al. 2015 [36]. In brief, we identified the probes that differed significantly between each individual cell type and PBMC using the Reinius et al. dataset (GSE35069) [37]. To calculate differential methylation, we used the linear regression approach (limma) with the following thresholds: *p* < 1e-07 and delta-beta > 0.05. No remarkable differences were identified before and after the filtering of the 8452 CpGs influenced by the white blood cell variability. Therefore, all the calculations in this manuscript were made without white blood cell variability correction. According to the study of J. H. Kim and colleagues [38], we excluded from our analysis four participants because of age or sex discrepancies. We selected 83 healthy males and 83 age-matched healthy females from the entire cohort (age range = 36–65). Out of 485,512 CpGs, 127,616 were excluded from our analysis for two main reasons: a) CpGs falling on sex chromosomes or on SNPs and b) CpGs showing cross-reactivity or polymorphisms [9, 39]. We chose the standard deviation as a measure to study CpGs inter-individual methylation variability (MV). Since our study was specifically focused on variability, we included only the CpGs with methylation values evaluated in all samples. Therefore, all the CpGs with missing values were not included in the analysis. At the end of the filtering process, 206,441 CpGs were obtained. To obtain MV values, we calculated mean, variance and standard deviation values of all samples for each CpGs. To associate each MV value to a CpG island density group, we used the association provided by Price and colleagues [9].

### Gene set enrichment analysis

To investigate the association between genes function and CpGs’ MV values, a Gene Set Enrichment Analysis (GSEA) was performed. We selected only the CpGs that fell in a range of 1500 base pairs before and after the Transcription Start Site (TSS) (− 1500, + 1500), and the resulting CpGs were associated with a gene according to Price criteria [9]. MV values of CpGs that were associated with the same gene were then mediated. In order to use the GSEAPreranked module on GenePattern [40], all the MV values were transformed in a z-score using this formula:

$$ \mathrm{z}=\left(\left(\mathrm{X}-\upmu \right)\right)/\upsigma $$

where z is the z-score, X is the MV value of that gene, μ is the population MV mean, and σ is the standard deviation of all the MV values. Using this linear transformation, we obtained positive and negative values that were sorted from the higher to the lower. The KEGG gene set (“c2.cp.kegg.v5.1.symbols.gmt [Curated]”) was used to perform the pathway analysis. We considered statistical significant all the genes sets that were enriched, at the same time, with a nominal *p*-value ≤0.01 and with a FDR q-val ≤ 0.25.

### Analysis of ancient selective pressure

To evaluate the presence of ancient selective pressure, we used a widely accepted conservation-based method: GERP-RS (Genomic Evolutionary Rate Profiling - Rejected substitutions) [12]. Detailed information about GERP-RS track can be found on UCSC website (http://genome.ucsc.edu). Briefly, the GERP-RS score estimates the conservation of each nucleotide in a multi-species alignment of 35 mammalians to the human genome 19 (hg19). For each base, it associates a “rejected substitutions” score that ranges from a maximum of 6.18 to a minimum of − 12.36. After downloading the entire GERP-RS track (GRCh37/hg19) from the UCSC database, we intersected GERP-RS values with CpGs position using BEDTOOLS [41], and we obtained a GERP-RS value for each CpG of our data set. GERP-RS scores can be interpreted as follows: a score near zero represents no substitution deficit and no signs of base conservation, while positive GERP-RS scores represent a substitution deficit, thus indicating that a base might be conserved. On the other hand, negative GERP-RS scores are difficult to be interpreted [12]. For this reason, we decided to remove all CpGs that were associated with a negative GERP-RS, therefore analyzing a total number of 80,098 CpGs with a GERP-RS score ≥ 0.

### Analysis of recent selective pressure

To evaluate the presence of recent selective pressure we used an ensemble of measures oriented to this aim. We extracted SNPs from the DataBase of recent Positive Selection across Human Populations (dbPSHP) [14], which contains lists of positive selected SNPs from the HapMap III and 1000 Genomes Project, filtered according to the following criteria:

-Derived Allele Frequency (DAF) > 0.05
-Genotype Frequency of Homozygous Derived Allele (GFHOM1) > 0.001
-Genotype Frequency of Heterozygote (GFHET) > 0.05
-*P*-value cutoff of Hardy-Weinberg equilibrium (HWE2) > 0.0001
-Heterozygosity (HET) < 0.5
-Nucleotide Diversity (PI) < 0.5
-Difference of Derived Allele Frequency (DDAF) > 0.2
-Tajima’s D (TD) < 0
-Fixation Index (FST1) > 0.05
-Integrated Haplotype Score (UIHS) > 1.5
-Cross-Population Extended Haplotype Homozygosity (UXPEHH) > 1
-Cross-Population Composite Likelihood Ratio (XPCLR) > 5

For more details, please see http://jjwanglab.org/dbPSHP. We used these criteria to download SNPs showing signals of recent selective pressure. Since the population included in our study is composed of people from Italy, we decided to use SNPs from populations with low genetic distance from our cohort. Therefore, only SNPs from TSI (Tuscany, Italy) and CEU (US residents with European ancestry) populations were included in the analysis. Since for each population, two different SNP datasets exist (1000 k genomes and HapMap III), they were merged, thus obtaining one final data set for each population. CpGs located in a range of 2000 base pairs around each filtered SNP were defined as Recent Selective Pressure-CpGs (RSP-CpGs). RSP-CpGs were identified using BEDTOOLS [41], obtaining 272 CpGs for TSI and 328 CpGs for CEU.

### Statistical analysis

All calculations, statistical analyses, and the bootstrapping estimations were performed using R statistical package version 3.2.5, with an alpha value set for *p* < 0.05.

## Additional file

Additional file 1: additional information about CEU population and bootstrap analyses. (PDF 557 kb)

## Abbreviations

dbPSHP: DataBase of recent Positive Selection across Human Populations
EPIC: European Prospective Investigation into Cancer and Nutrition
GERP-RS: Genomic Evolutionary Rate Profiling - Rejected substitutions
GSEA: Gene Set Enrichment Analysis
MV: inter-individual methylation variability
TSS: Transcription start site
