# Supplementary material for: DNA Methylation variability among individuals is related to CpGs cluster density and evolutionary signatures
Source: BMC Genomics. 2018 Apr 2;19:229. doi: 10.1186/s12864-018-4618-9 (PMC5880022; doi:10.1186/s12864-018-4618-9)
Supplement: Supplementary file 1 — additional information about CEU population and bootstrap analyses. (PDF 557 kb) [file 12864_2018_4618_MOESM1_ESM.pdf]

Additional file 1

Many measures aimed to identify signatures of recent selective pressure have been described (Li et al. 2014) and they are based on SNPs frequencies. To select SNPs under recent selective pressure, we used different cutoff on several selective signals (as described in Materials and Methods section). We arbitrary denoted as region under recent selective pressure 2000 base pair around each SNP and we called “Recent Selective Pressure-CpGs” (RSP-CpGs) the CpGs that fell in these regions. Since recent selective signals are population specific, we selected only SNPs under recent selective pressure in TSI (Tuscans in Italy) and CEU (Utah Residents with Northern and Western Ancestry) because genetically less distant from our dataset. Table S1 shows the number of SNPs selected from dbPSHP (http://jjwanglab.org/dbpshp).

Table S1.

SNPs and CpGs selected from dbPSHP

Project name	SNPs	RSP-SNPs	RSP-CpGs
1KGP TSI	782	73	143
HapMap TSI	688	74	191
1KGP CEU	923	67	122
HapMap CEU	762	83	238

From left: Project name (1000 Genome Project and HapMap), SNPs (number of SNPs for that specific population and project), RSP-SNPs (SNPs under recent selective pressure in that specific population and project) and RSP-CpGs (recent selective pressure CpGs in that specific population and project).

The CpGs located within these regions were selected with BEDTOOLS (Quinlan and Hall 2010). The two different datasets for each population (1000k genomes and HapMap III) were merged, obtaining one final dataset for each population (272 CpGs for TSI and 328 CpGs for CEU). RSP-

CpGs show lower MV values than the others do (TSI MV mean = 0.027, No TSI MV mean = 0.035; CEU MV mean = 0.031, No CEU MV mean = 0.035) (Additional figure S1).

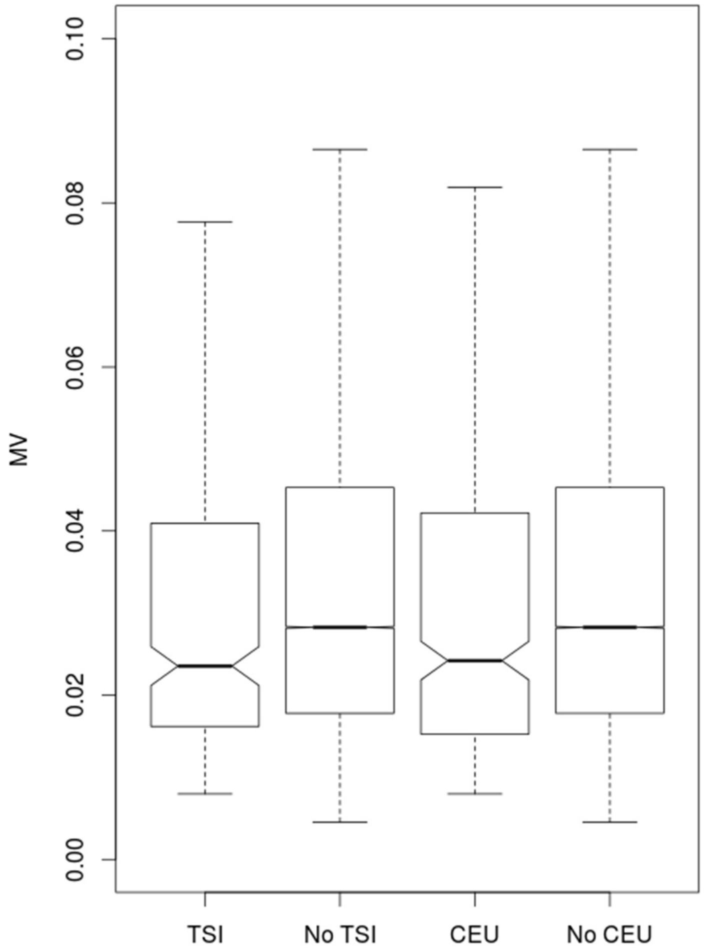

Additional figure S1. Boxplots of MV values in TSI, CEU and corresponding No RSP-CpGs regions ("No TSI" and "No CEU").

To test whether there was a statistically significant difference between RSP-CpGs MV and the remaining CpGs (No TSI and No CEU), we performed a Bonferroni corrected t-test analysis on MV values of TSI and CEU RSP-CpGs and on MV values of No TSI and No CEU. It revealed that all pairwise comparisons result significant at p-value $< 1.4 \times 10^{-10}$. Because the two datasets (CEU and TSI) differ for length from the No RSP-CpGs datasets, we also performed a bootstrapping analysis

that confirmed the previous results (bootstrap analysis based on 10000 Monte Carlo simulations, TSI = p-value < 1 x 10^{-4}, CEU = p-value < 3 x 10^{-3}) (Additional figure S2).

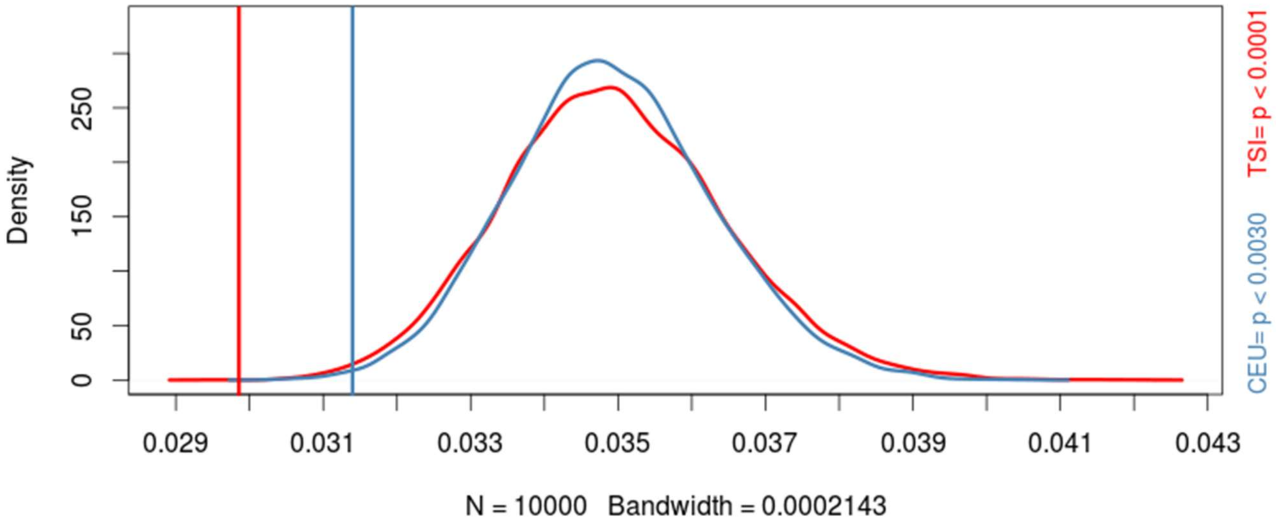

Additional figure S2. Bootstrapping density plot of 10000 random resampling of MV values from EPIC Italy dataset. Blue and red curve represent 10000 MV values using CEU population size (328) and TSI population size (272) respectively. The blue line represents mean MV of Recent Selective Pressure CpGs (RSP-CpGs) in CEU population, and the red line represents mean MV of RSP-CpGs in TSI population. P-values are on the y-axis on the right.

In particular, RSP-CpGs MV values were compared with 10000 samples of the same population size (272 CpGs for TSI and 328 CpGs for CEU), randomly extracted from the population of No RSP-CpGs.
